# The search for charmed states of matter

**DOI:** 10.1093/nsr/nwab008

**Published:** 2021-01-20

**Authors:** Philip Ball

**Affiliations:** NSR, London

## Abstract

With the discovery of the Higgs boson in 2012, the Standard Model of particle physics was completed. This description encompasses all known subatomic particles and their interactions. Much of the public interest in high-energy physics now focuses on experimental searches for ‘new physics’ beyond the Standard Model. Yet it would be a mistake to imagine that the Standard Model is now fully understood. Many questions remain about the ways in which known fundamental particles interact and unite, especially at the very high energies needed to produce the most exotic varieties, such as heavy quarks (quarks are the constituents of hadrons, which include the ordinary nuclear particles protons and neutrons) and heavy leptons (leptons are members of the family that includes electrons).

The Beijing Electron-Positron Collider (BEPC), operated by the Chinese Academy of Sciences' Institute of High Energy Physics, is one of the installations that are probing these questions. It has been running since 1988, using a detector called the Beijing Spectrometer (BES). (The site also houses the Beijing Synchrotron Radiation Facility for conducting studies in condensed matter using intense X-rays.) Since 2008, these two instruments have been operating in upgraded form: the BESIII detector and BEPCII accelerator. The facility is now one of the key international centers for investigating the properties and behavior of new exotic hadrons, in particular those that include the charm quark. Italian physicist Luciano Maiani, Director of the European particle physics center CERN in Switzerland from 1999 to 2003, is one of the world leaders in this area of high-energy physics, and played a central role in the identification of the charm quark itself. NSR spoke to him about the aims of the latest work at the BEPCII, and the prospects for new discoveries.

***NSR*:** What are the main improvements that were made for BESIII and BEPCII, and what new regimes of energy, beam intensity and/or sensitivity do they access?

***Maiani*:** BEPCII is a particle accelerator of a special kind called a collision ring. The first of these devices was realized by Austrian physicist Bruno Touschek in Italy in the 1960s. In this machine, two beams of particles, one with electrons (e^−^) and the other with positrons (e^+^, the electron's antiparticle), are accelerated and kept in two circular orbits under extreme vacuum. Where the orbits intersect, electrons and positrons collide head on. In a few cases, an electron and a positron interact so closely that they annihilate one another, giving rise to a wealth of subatomic particles that can be studied by appropriate particle detectors. BESIII is one such detector, and measures the energy, direction of flight, electric charge and other physical properties of the particles created in the annihilation event, thus identifying their nature and the correlations among them. Collision rings are characterized by the beam energy, which determines the maximum mass of the particles produced, and the luminosity, related to the density of particles in the beam and which determines the collision rate.

BEPC can produce particles containing a pair of charm quarks, such as the *J*/Ψ meson discovered by Burton Richter and Samuel Ting (which won them the 1976 Nobel Prize). Particle detectors are characterized by the precision with which particle energies can be measured, the energy resolution, and by their capability for detecting neutral particles such as photons, which are an important tool for identifying new particles produced in *e*^+^*e*^−^ annihilation. The labels II and III indicate progressive increases in luminosity (for BEPC) and in energy resolution and neutral particle detection (for BES), with respect to the original design.

**Figure:**
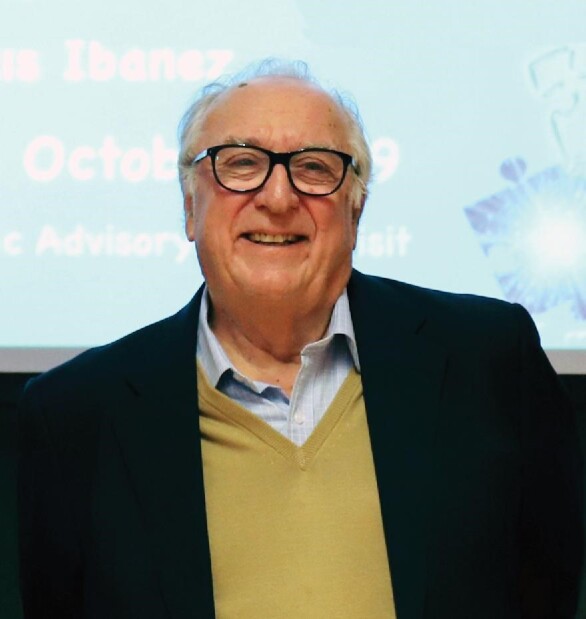
Italian physicist Luciano Maiani, Director of the European particle physics center CERN in Switzerland from 1999 to 2003 *(courtesy of Luciano Maiani)*.

Among facilities of this kind, BEPCII currently has the highest luminosity in the world.

—Luciano Maiani

***NSR*:** How, in short, does BESIII work?

***Maiani*:** The annihilation of an *e*^+^*e*^−^ pair can produce a single unstable particle, called a ‘resonance’, which appears as a bump in the annihilation probability as a function of beam energy (that is the ‘spectroscopy’), with a width inversely proportional to the particle's lifetime. (The amount of this indeterminacy in energy is inversely related to the lifetime of the particle via Heisenberg's Uncertainty Principle.) In this way, we can determine lifetimes of the order of 10^−20^–10^−23^ seconds, which is something we could never assess from direct measurements of this timescale. That is how the *J*/Ψ particle and many other charm-anticharm states were first observed in the 1970s at the SLAC collider in Stanford, California.

As well as the resonance, the annihilation produces many other particles. However, particular clusters of the final particles may themselves arise from the decay of an unstable particle. To identify the parent particle, one plots the distribution of the total mass of the cluster. A bump in this distribution, and the corresponding width, gives the mass and lifetime of the parent resonance. In this way, BESIII has discovered the resonances denoted *Z*_C_(3900) and *Z*_C_(4020), where the numbers indicate the masses in MeV. These resonances are made of a pair of charm quarks accompanied by a pair of lighter quarks, as indicated by the fact that they are electrically charged (unlike the *J*/Ψ). These *Z*_C_ resonances are among the first examples of subnuclear particles that require, in their constitution, at least two quark–antiquark pairs. The *Z*_C_^+^, for example, is made from a $c\bar{c}$ and a $u\bar{d}$ pair [Here *c*, *u* and *d* denote the charm, up and down quarks, and the bars indicate their antiparticles.].

***NSR*:** Are there any other instruments in the world that could perform experiments like those at BESIII? How do its capabilities compare with those at CERN, for example?

***Maiani*:** The particles studied with BESIII are in the range of particles made by a $c\bar{c}$ pair (called charmonia) plus, eventually, other constituents. These particles are called hidden charm particles, because they have zero net charm quantum number: the charm and anticharm quarks ‘cancel out’. Hidden charm particles are produced in high-energy hadron colliders like the Large Hadron Collider (LHC) at CERN—but there they feature in events with a large background of other particles, and so it is hard to see the resonances. Low background is a crucial feature of *e*^+^*e*^−^ colliders like BEPCII.

Among facilities of this kind (called charm-tau factories), BEPCII currently has the highest luminosity in the world. A lower-luminosity machine, VEPP2000, is working at the Budker Institute in Novosibirsk, Russia, with plans for a considerable upgrade in the coming years. At higher energy, the $b\bar{b}$ factory at KEK in Tsukuba, Japan, with the Belle detector, can reach the hidden charm range by restricting the observation of events to those in which one of the initial particles (the electron or positron) loses energy by radiating a photon so as to bring the center-of-mass energy of the annihilation into the charm-tau range.

The Belle and LHCb collaborations have produced valuable results on exotic hadrons. But as far as resolution and luminosity are concerned, BEPCII and BESIII are firmly in the forefront of global research on hidden charm particles.

## TESTING THE THEORY OF QUARK BINDING

***NSR*:** There is a well-established theory—quantum chromodynamics (QCD)—for describing the properties of ‘light’ hadrons in terms of the interactions of light quarks and gluons. And yet it seems that there are still questions to be asked about this sector of high-energy physics. What are the key issues here?

***Maiani*:** QCD has been tested in phenomena such as high-energy, large-angle scattering of electrons off protons (called deep inelastic scattering). In these conditions, the coupling that regulates the QCD interaction is small—this property is called asymptotic freedom, the discovery of which won David Gross, David Politzer and Franck Wilczek the 2004 Nobel Prize. In these conditions the interaction can be treated in close analogy to quantum electrodynamics (the quantum description of matter–light interactions), and the theory satisfactorily describes the experimental results. But the binding of quarks into baryons and mesons (hadrons) puts QCD in the very strong interaction regime, and the connection of the fundamental theory to the details of the binding is not (yet) well-established. This makes hadron spectroscopy of great interest, because those experiments can give us clues about how to build a theory of the bound states, in particular to determine the dominant forces binding multiquark mesons such as the resonance *Z*_C_^+^, in comparison to the simplest $c\bar{c}$ states (that is, charmonia). Multiquark spectroscopy is thus the new, largely unexplored, frontier of quantum chromodynamics.

***NSR*:** There seem to be some exotic particles and states predicted in the low-energy light-hadron regime, such as ‘glueballs’. What are these, and why are they important?

***Maiani*:** In QCD, the strong interactions are transmitted by gluons: massless particles with spin 1, analogous in many respects to the photons that transmit the electromagnetic force, except that they are able to interact strongly with each other. As a consequence, one can conceive of bound states called ‘glueballs’ made of gluons only, which would be neutral under all possible elementary particle symmetries. Theoretically, it is difficult to distinguish gluons from quark–antiquark states in which the quarks are arranged so as to neutralize all possible quantum numbers related to symmetry: these are called ‘singlet’ configurations. At the moment, among the observed resonances, there are few cases where more singlet particles have been seen than are

Multiquark spectroscopy is the new, largely unexplored, frontier of quantum chromodynamics.

—Luciano Maiani

predicted by the quark model—and so these can be considered to be candidates for these elusive glueballs.

***NSR*:** One of the primary goals of BESIII seems to be to probe the physics of heavy quarks such as the charm quark. You yourself played a key role in the discovery of this particle. Can you tell us how that came about?

***Maiani*:** If hadrons are made of three quark types only (up, down and strange), as originally proposed by Murray Gell-Mann and George Zweig in 1963, it implies that the weak force would be mediated by a particular boson that produces the so-called Cabibbo transition, by which a *u* quark and the weak-force particle *W*^−^ interconvert with a particular combination of *d* and *s* quarks introduced by Nicola Cabibbo. Attention was focused in 1968 on the so-called ‘neutral current processes’, which are forbidden in the first approximation but can be generated by including ‘corrections’ with amplitudes that can in principle grow without limit. In 1970, Sheldon Glashow, John Iliopoulos and I (collectively, GIM) proposed that these processes might involve a fourth quark, designated ‘charm’ (*c*). (Such a quark had already been suggested by others for completely different reasons.) This idea turned a puzzle in the three-quark theory into a way of estimating the mass of this putative fourth quark.

The predicted *c* quark mass was sufficiently large to explain the unsuccessful searches for mesons containing it that had been conducted in the 1960s. The GIM mechanism has been an important step towards a unified theory of the electromagnetic and weak interactions, allowing hadrons (governed by strong interactions) to be included in the picture. The existence of the charm quark was confirmed by the discovery of the *J*/Ψ particle in 1974.

## THE WORLD OF CHARMONIA

***NSR*:** In charm physics, the notion of ‘charmonium’ states seems to play a central role. What is this?

***Maiani*:** Calculations of the neutral-current processes that motivated the GIM mechanism have been carried out in the electroweak theory and have confirmed the large mass of the charm quark: *M*_C_ ≈ 1.8 GeV. With the advent of QCD, the large value of charm mass appeared in a different light. It is known that electron–positron pairs form bound states known as ‘positronium’. In 1974, Thomas Appelquist and David Politzer considered the analogous state formed by a $c\bar{c}$ pair bound by QCD forces, which they called ‘charmonium’. The idea was taken up by Sheldon Glashow, Alvaro de Rujula and Howard Georgi, to explain the surprisingly narrow width of the just-discovered *J*/Ψ, which implied that that particle might be the first manifestation of the charm quark (in hidden-charm disguise). In the following years, many authors presented accurate quantitative calculations of the numerous charmonia states discovered in electron–positron colliders after the *J*/Ψ . That success was repeated at higher mass, after the discovery of similar resonances of $b\bar{b}$ quark pairs.

***NSR*:** There seems to be a dizzying array of possible particles, resonances and transitions involving charm quarks. Can you help us navigate this ‘zoo’ by explaining what some of the key issues are?

***Maiani*:** Heavy quark pairs are difficult both to create and to destroy by QCD forces. The first examples of such exotic hadrons were resonances whose decay products contain a charmonium (and therefore a $c\bar{c}$ pair) but do not fit the spectrum of charmonia accurately computed by QCD. Such resonances have been dubbed ‘unanticipated charmonia’ and classified, provisionally, as *X*, *Y* and *Z* states. The first unanticipated charmonium, *X*(3872), was found by the Belle collaboration in Japan in 2003; this state decayed into *J*/Ψ and pion particles. *X*(3872) cannot be a charmonium state, however, because its mass does not fit with predictions and because its pion decays do not follow the rules obeyed by pure charmonia. A second unanticipated resonance, *Y*(4260), was found by the Babar experiment at SLAC, with a mass that also does not fit the charmonium spectrum. The first example of electrically charged unanticipated charmonium, *Z*(4430), was found by Belle in 2007, but its existence as a genuine resonance was put in doubt by Babar. However, in 2014 the LHCb instrument at CERN, with improved statistics, confirmed *Z*(4430) as a genuine resonance.

In 2013, as indicated earlier, BESIII discovered two other charged resonances with charmonium decay: *Z*_C_(3900) (which decays into *J*/Ψ and a pion) and *Z*_C_(4020) (which decays into a pion and a charmonium state with the same spin as *J*/Ψ but opposite parity, denoted *h*_c_). Over the past 10 years, BESIII has produced a wealth of results on the *Y* states, including analogies among the *X*(3872), *Y*(4260) and *Z*_C_(3900) states.

Even so, no consensus has been reached yet about how quarks are organized inside *X, Y* and *Z* resonances. One possibility is the ‘compact tetraquark’, where a diquark (*cq*) is bound to an anti-diquark ($\bar{c}{\bar q^{\prime}} $) by QCD forces, similar to those that bind $c\bar{c}$ into a meson. With improved statistics and resolution, BESIII might be able to help distinguish among this and other models.

## THE SEARCH FOR NEW PHYSICS

***NSR*:** What are the prospects for discovering new physics (that is, physics beyond the Standard Model) at BESIII? Can you speculate on what this might be? Is there any prospect, for example, of shedding new light on dark matter, or on the origin of the asymmetry in the amounts of matter and antimatter in the universe?

***Maiani*:** One fascinating proposal is that dark matter consists of new particles not coupled to the forces of the Standard Model.

BESIII could perhaps even discover a signature of dark photons.

—Luciano Maiani

There could be a new photon, called a ‘dark photon’, for which all known particles have zero charge (meaning that they do not interact with it). But by a quantum-mechanical effect, this dark photon could spend a small fraction of its time as a normal photon, thus acquiring a small coupling to, for example, electrons and muons. That would mean a small fraction of the particles produced by an *e*^+^*e*^−^ collider could be dark photons that would produce anomalous signals, for example in the spectrum of *e*^+^*e*^−^ pairs in the final state. Previous low-energy colliders have set limits on the coupling and mass of dark photons. BESIII could extend considerably the regime that could be explored for such coupling, and perhaps even discover a signature of dark photons.

Matter/antimatter symmetry is violated both in fundamental interactions and in the universe at large scales—the Sun, for example, is made of matter, and there is no evidence of anti-stars or anti-galaxies made of antimatter. We do not know if these two asymmetries are related, or whether one can explain the latter by the former. BESIII may shed light on this fundamental question by measuring the matter/antimatter asymmetries in the weak decays of charmed mesons, by comparing the asymmetry in charm decays to the known asymmetries in strange and beauty mesons. This is largely unexplored at the moment.

## COLLABORATIONS IN CHALLENGING TIMES

***NSR*:** This is evidently a highly international collaboration, albeit with a strong Chinese representation. As a previous Director General of CERN, you will doubtless know very well the challenges of maintaining such a vast project. What are these? And have they become more complicated in the age of Covid-19 and uncertainties about travel?

***Maiani*:** The IHEP has a very good record in assembling and running complex experiments carried out by international collaborations. Experiments like BESIII work with the same kind of rules as CERN experiments, albeit on a smaller scale. Just as at CERN, Covid-19 is creating difficulties for on-site collaborations. But external groups currently confined to their own countries can still contribute to some degree, for example with data analysis, conducting simulations of device performance, and so on. Even a partial resumption of international travel, as is now happening at CERN, would be of great help.

***NSR*:** How, if at all, does research in this field differ in China compared to, say, the US, Europe and Japan? Does each region have its own unique ‘flavor’?

***Maiani*:** Over the last four years, I spent a good part of my time working in Beijing (Institute of High Energy Physics—IHEP) and Shanghai (Shanghai Jiao Tong University). Except for obvious (and sometimes exciting) differences in lifestyle, food and so on, I can see that science is really universal in scope, method and sources of inspiration.

***NSR*:** How can researchers, especially young researchers, hope to make their mark in projects that require such huge teams? Is a different ethos required, in which scientific understanding is seen to be the product of selfless teamwork as opposed to the individualistic approach often seen in other fields?

***Maiani*:** One has to see a large collaboration such as BESIII as a ‘laboratory’ of its own, which offers to small teams different fields of research (instrumentation, precision measurements, data analysis, phenomenology and so on). Inside each team, individual talent, skill and ingenuity can shine and be appreciated. A young post-doc can show to senior old hands the way to solve their current problem. In this way, like in the old times, a young person may acquire a reputation that will bring her or him to larger responsibilities inside the ‘laboratory’ and beyond. This is the path by which Fabiola Gianotti and Yifang Wang have become the directors of CERN and IHEP.

